# Expression of connexin 43 protein in cardiomyocytes of heart failure mouse model

**DOI:** 10.3389/fcvm.2022.1028558

**Published:** 2022-10-05

**Authors:** Shaoyan Liu, Yang Lan, Yun Zhao, Qianyu Zhang, Tzuchun Lin, Kaibin Lin, Junjie Guo, Yan Yan

**Affiliations:** ^1^Department of Cardiology, Zhongshan Hospital, Fudan University, Shanghai, China; ^2^Department of Cardiac Surgery, Zhongshan Hospital, Fudan University, Shanghai, China; ^3^School of Life Sciences and Technology, Shanghai Tech University, Shanghai, China; ^4^Department of Cardiology, The Affiliated Hospital of Qingdao University, Qingdao, China; ^5^Qingdao Municipal Key Laboratory of Hypertension (Key Laboratory of Cardiovascular Medicine), Qingdao, China

**Keywords:** heart failure, aortic valve stenosis, Cx43, *Cx43-BFP-GFP*, mouse model

## Abstract

Heart failure (HF) is the end stage of various cardiovascular diseases, with high morbidity and mortality, and is associated with a poor prognosis. One of the primary causes of HF is aortic valve disease, manifested by progressive aortic valve stenosis (AVS), resulting in increased left ventricular load, ventricular hypertrophy, ultimately ventricular dysfunction, and HF. Early assessment of the degree of cardiomyopathy and timely intervention is expected to improve patients’ cardiac function and delay or even avoid the occurrence of HF. The Wnt signaling pathway is mainly involved in regulating myocardial insufficiency after valve stenosis. Connexin 43 protein (Cx43) is an essential target of Wnt signaling pathway that forms gap junction (GJ) structures and is widely distributed in various organs and tissues, especially in the heart. The distribution and transformation of Cx43 among cardiac cells are crucial for the development of HF. To specifically label Cx43 *in vivo*, we established a new *Cx43-BFP-GFP* mouse model with two loxp sites on both sides of the tag BFP-polyA box, which can be removed by Cre recombination. This double-reporter line endowed us with a powerful genetic tool for determining the area, spatial distribution, and functional status of Cx43. It also indicated changes in electrical conduction between cells in a steady or diseased state.

## Introduction

Heart failure (HF) is a chronic disease associated with poor outcomes and high mortality rates (1). It is the main cause of non-elective hospitalization in developed countries for patients over 65 years of age (2). HF is caused by myocardial damage for various reasons, of which heart valve disease is one of the major causes (3). Aortic valve stenosis (AVS) is an independent factor in left ventricular failure (4). Roughly 10% of patients with AVS suffer from heart failure with reduced ejection fraction (HFrEF) (5), and people with this type of HF are more likely to have cardiovascular accidents. The determinant of cardiac function improvement after aortic valve replacement (AVR) in AVS patients is the degree of preoperative myocardial fibrosis (6). Fibrotic remodeling of the myocardium disrupts normal myocardial excitation-contraction coupling, resulting in cardiac dysfunction (7). It is considered one of the essential factors in the resulting HF (8, 9).

The most common communication between adjacent cells is through gap junction (GJ) channels composed of connexins (10). GJ channels mediate electric communication between cardiomyocytes to coordinate contraction (11). The heart’s three major interstitial GJ proteins are Cx40, Cx43, and Cx45 (12). The Gja1 gene found on human chromosome 6, codes for the protein Cx43 (13). Cx43 is ubiquitously distributed in various tissues and organs of mammals, being the most abundant in the heart (14, 15). Previous research showed that decreased Cx43 expression is associated with excessive fibrotic deposition, ventricular dysfunction (16, 17) and weakened GJs at cardiac intercalated discs (18). The Wnt signaling pathway is significantly involved in the pathogenesis of cardiac fibrosis (19). Recent studies have reported that Cx43 is a novel target protein activated in response to extracellular Wnt signaling (20).

Although an essential protein for indicating and regulating heart functions, the lineage tracing and cell-to-cell transformation of Cx43 in various cardiac cells, especially cardiomyocytes, has always been challenging due to the lack of efficient tools. Thus, to specifically label Cx43 *in vivo*, we present a new *Cx43-BFP-GFP* mouse line by inserting the loxp-tagBFP-WPRE-polyA-loxp-eGFP-WPRE-polyA cassette into 3′-UTR of the Cx43 gene by homologous recombination. The expression of blue fluorescent protein (BFP) and green fluorescent protein (GFP) can be regulated by independent recombination events without affecting each other. The heterozygous mice carrying the knock-in allele are healthy and fertile. Therefore, the *Cx43-BFP-GFP* reporter knock-in mouse line provides a unique genetic tool for understanding the role of Cx43 in disease homeostasis and pathological processes. In addition, exploring the distribution of GJs in cardiomyocytes caused by valve stenosis in the mouse model may provide insights into a better understanding of the biological mechanism behind HF, facilitating timely intervention to delay or avoid the development of HF in medical practices.

## Materials and methods

### Human myocardium samples

Myocardial tissues were collected from three patients who underwent heart transplantation at Zhongshan Hospital Affiliated with Fudan University. Patients with diabetes, hypertension, rheumatic valvular disease, and other cardiomyopathy were excluded from this study. Written informed consent was obtained from each patient before surgery with the approval of the Ethics Committee of Zhongshan Hospital, Affiliated with Fudan University (Ethical approval number: B2022-031R). Tissues were collected during surgery, and patient’s medical records were reviewed to assess clinical data and were performed in accordance with the Declaration of Helsinki.

### Experimental animals

The animal study was reviewed and approved by the Ethics Committees of Zhongshan Hospital, Affiliated with Fudan University for the Care and Use of Animals for Research Purposes. These mouse lines were generated by Shanghai Biomodel Organism Science and Technology Development, Shanghai, China. Mice were maintained in a C57BL/6J genetic background and bred with a normal diet.

For generation of the *Cx43-BFP-GFP* mouse line, the CRISPR/Cas9 technology was used to insert loxp-tagBFP-WPRE-polyA-loxp-eGFP-WPRE-polyA cassette at the start codon site of Gja1 gene through homologous recombination. The brief process was as follows: Cas9 mRNA and gRNA were obtained by *in vitro* transcription. Homologous recombination vector was constructed by in-fusion cloning method, which contains a 3.5 kb 5′ homology arm, loxp-tagBFP-WPRE- polyA-loxp-eGFP-WPRE-polyA, and 3.2 kb 3′ homology arm. Cas9 mRNA, gRNA, and donor vector were microinjected into the fertilized eggs of C57BL/6J mice to obtain F0 generation mice. The positive F0 generation mice identified by PCR amplification and sequencing were mated with C57BL/6J mice to obtain positive F1 generation mice for the experiment.

### Genomic polymerase chain reaction

Genotyping of mice was achieved by PCR amplification. Genomic DNA was extracted from the toes of mice. These toes were digested by lysis buffer with proteinase K at 55°C oven overnight, followed by centrifuging at 15,000 rpm for 8 min to obtain the supernatant, added with isopropanol to precipitate the DNA, and then washed in 70% ethanol twice. The PCR primers used to identify wild-type mice were 5′-AGTTGGGCTCGCTCTTCTCC-3′ and 5′ TGCCGTGTTCTTCAATCCCATACT-3′ (wild-type allele). For the *Cx43-BFP-GFP*/ + line, we used the genotyping primers 5′-GATTTGCCCTTGGATTCTGTTTTG-3′ and 5′-CGCCCCCGTCTTCGTATGT-3′ to detect.

### Tissue whole-mount fluorescence microscopy

Isolated organs and tissues were washed several times in PBS to clear blood, and the excess tissues were removed under the bright field of the microscope. Then placed on transparent agar in an anatomical direction to obtain the bright-field whole-mount images and fluorescent images by the Zeiss stereoscope (AxioZoom V16). We applied the automatic z-stack image function by the Zeiss stereoscope (AxioZoom V16) to obtain the magnification of a specific area.

### Tissue collection and Immunofluorescence staining and imaging

The isolated organs were fixed in 4% paraformaldehyde (PFA) for 1 h and washed 3 times in PBS, then placed them in 30% sucrose solution to dehydrate at 4°C overnight. The samples were embedded and frozen in OCT compound (Sakura, 4,583) for freezing and then stored at –80°C. For immunofluorescence staining, the frozen sections were cut into 10 μm thick and placed in a fume hood for 30∼60 min. Subsequently, the slices were blocked with 5% normal donkey serum and 0.1% Triton X-100 PBS for 30 min at room temperature after 3 times of PBS washing. Then sections were incubated with primary antibodies overnight at 4°C. After that, sections were incubated with corresponding secondary antibodies for 40 min at room temperature in the dark after 3 times of PBS washing. After three washes, the stained sections were fixed with 50% glycerol and stored for further analysis. The immunostained images were obtained by Zeiss confocal microscope system (LSM710) and Olympus laser scanning confocal microscope (Fluoview 1200). The primary antibodies used for immunofluorescence staining were as follows: Cx43 (Sigma-Aldrich, C6219, 1:100); α-SMA (Sigma, F3777, 1:100); Cdh5 (R&D, AF1002, 1:100); PDGFRα (R&D, AF1062, 1:100); Troponin I (Abcam, ab56357, 1:100); CD45 (eBioscience, 17-0451-82, 1:100); DAPI (Vector Laboratories, 1:1,000). Used Alexa-conjugated secondary antibody (Invitrogen) at a concentration of 1:1,000.

### Hematoxylin-esosin staining

Tissue slices were soaked in PBS for 10 min and then placed in hematoxylin for 5 min, and placed in hydrochloric acid ethanol for 1 min after rinsing with running water. Next, put the sections in ammonia water for 5 min after dyeing, then in 95% ethanol for 1 min, eosin dye for 8 s, and rinsed in deionized water. Subsequently, placed the stained slices in 95% ethanol for 1 min, 100% ethanol for 4 min, and xylene for 10 min. Mounted the slices with mounting medium.

### Sirius red staining

Tissue slices were soaked in PBS for 15 min and then fixed in 4% PFA for 15 min, then washed 3 times with PBS. Next, the cryo sections were incubated overnight at room temperature in Bouins’ solution, then washed 3 times in PBS. It was then sequentially incubated in 0.1% Fast Green for 5 min and 0.1% Sirius red for 3 min. The slides were sequentially dehydrated in ethanol for 5 min and in xylene for 5 min after being washed 3 times in PBS. Finally, mounted the slices with mounting medium.

### Transverse aortic coarctation

The mice were placed in a container containing isoflurane (2% isoflurane dissolved in 100% O_2_, 0.8–1.2 L/min) for anesthesia and then fixed on the operating table. Then the mice were connected to an animal ventilator at 120–140 times/min and 0.2 mL tidal volume after endotracheal intubation. Thoracotomy was performed on the second rib under a microscope after disinfecting the surgical site with 75% alcohol. Passed the 6.0 thread through the aortic arch between the innominate artery and the left carotid artery, placed a 26 G pad over the intended ligation site of the aortic arch, then wrapped the ligature around the aorta and ligate with a 26 G pad, and carefully withdraw the pad. Closed the chest after confirming that there was no hemothorax or pneumothorax. Removal of the tracheal tube when mice breathe spontaneously.

### Animal echocardiography

Depilated the mice before the procedure. The mice were placed in a container containing isoflurane (2% isoflurane dissolved in 100% O_2_, 0.8–1.2 L/min) for anesthesia, and then fixed on the operation board of a cardiac ultrasound machine to expose the chest. Then the mice limbs were connected to sensing electrodes and added conductive liquid to record ECG signals. Data collection was performed by a professional echocardiographer. Echocardiography was conducted with the VisualSonics Vero 2100 system. The measurement parameters included ejection fraction (EF), fractional shortening (FS), left ventricular internal diameter at end-diastole (LVIDd), and left ventricular internal diameter at end-systole (LVIDs).

### Quantification and statistical analysis

The obtained images were processed and analyzed using PhotoLine software. Statistical analyses were performed using Student’s *t*-test with GraphPad Prism 8 software. All data were representative of at least three independent experiments and presented as mean ± SD. Student’s tests were applied for comparison between two groups. *p-value of < 0.05, ^**^p-value of < 0.01, ^***^p-value of < 0.001, and ^****^p-value of < 0.0001 were considered as statistically significant difference.

## Results

### Myocardial characteristics of aortic valve disease

**FIGURE 1:**
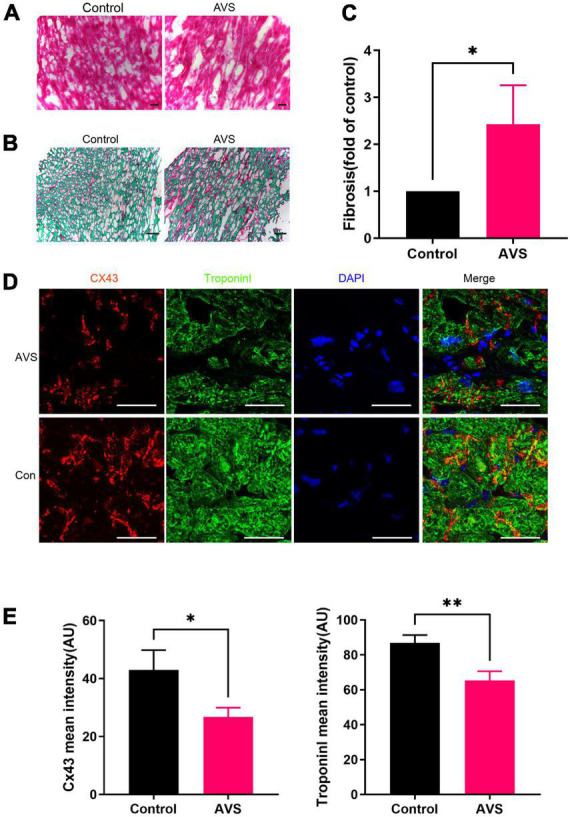
Myocardial characteristics of aortic valve disease. **(A)** HE staining of myocardial tissue sections. **(B,C)** Sirius red staining of myocardial tissue sections and its quantification analysis, fast green as background color, *P < 0.05; **(D)** immunofluorescence staining of Cx43 (red) and Troponin I (green) in the myocardium. Scale bar = 100 μm, AVS, Aortic Valve Stenosis; Con, Control; **(E)** remarkably fading of Cx43 and Troponin I fluorescent intensity were observed in AVS myocardium. *P < 0.05, ^**^P < 0.01.

Immunohistochemical results showed that cardiomyocytes in patients with severe AVS were abnormally hypertrophic and disordered, with uneven cytoplasmic staining and decreased intercellular density compared with normal myocardial tissues (Figure 1A). In addition, Severe myocardial collagen deposition in AVS patients. The fibrosis area in AVS patients was approximately 2.5 times higher than that of the control group (Figures 1B,C). Progressive myocardial fibrosis in aortic stenosis drives the progression of HF (21). Therefore, we stained myocardial tissues for Cx43 and the cardiomyocyte-specific marker protein Troponin I and found that Cx43 expression was significantly reduced and unevenly distributed in AVS myocardium compared with normal myocardium (Figure 1D). The fluorescence intensity of both Cx43 and Troponin I decreased by more than 20% compared to the control ones (Figure 1E). Information of patients and donors is provided in Supplementary Table 1.

### Generation of *Cx43-BFP-GFP* reporter line

**FIGURE 2:**
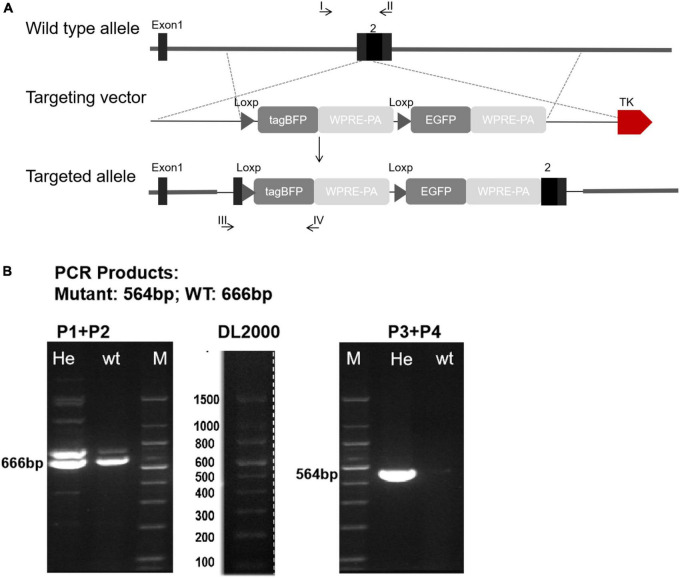
Generation of *Cx43-BFP-GFP/* + reporter line. **(A)** Schematic diagram showing the targeting strategy of *Cx43-BFP-GFP* allele by homologous recombination using CRISPR/Cas9; **(B)** polymerase chain reaction (PCR) of genomic DNA, P1 + P2 means primer1 + primer2; P3 + P4 means primer3 + primer4; Heterozygous amplified a 564 bp band.

In order to further explore the distribution and expression of Cx43 in valvular stenotic cardiomyopathy, we constructed a tool mouse *Cx43-BFP-GFP* to specifically label Cx43. To generate *Cx43-BFP-GFP* knock-in mouse line, we inserted a loxp-tagBFP-WPRE-polyA-loxp-eGFP-WPRE-polyA cassette into the 3′-UTR of the Gja1 gene through homologous recombination. In the targeting construct, the tagBFP and polyA stop cassette (tagBFP-polyA) is flanked by two loxp sites, and the enhanced GFP is followed by a second loxp as illustrated in Figure 2A.

To optimize the genotyping protocol for *Cx43-BFP-GFP* knock-in, we re-designed four PCR primers [P1 and P2 to amplify the wild-type allele (666 bp) and P3 and P4 to amplify the inserted site (564 bp)], which were located outside or inside of the loxp-tagBFP-WPRE-polyA-loxp-eGFP-WPRE-polyA cassette sequence (Figure 2B). Genotyping by PCR using the forward and reverse primers showed that the loxp-tagBFP-WPRE-polyA-loxp-eGFP-WPRE-polyA cassette was successfully inserted into the Cx43 locus. No defects were observed in the generated heterozygous *Cx43-BFP-GFP* reporter mice, compared with wild-type littermates.

### Characterization of *Cx43-BFP-GFP* reporter line at 6 weeks

**FIGURE 3:**
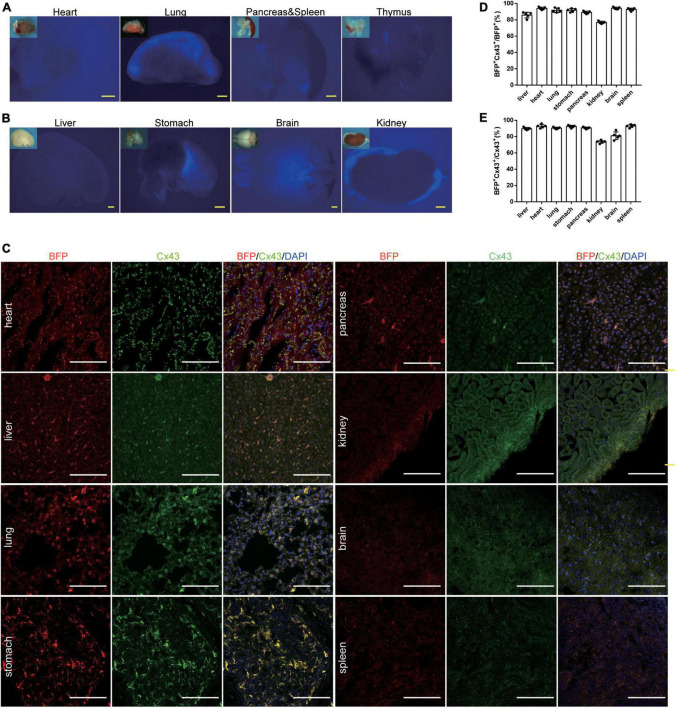
Generation and Characterization of *Cx43-BFP-GFP/* + reporter line. **(A,B)** Whole-mount bright field and epifluorescence images of visceral organs from 6-weeks *Cx43-BFP-GFP/* + mice; **(C)** immunostaining for BFP and Cx43 on heart, lung, brain, stomach, kidney, liver, spleen, and pancreas sections. The high magnification images of organs in areas labeled with the square dotted frame, respectively; **(D)** quantification of the labeling efficiency by the percentage of BFP^+^ Cx43^+^ in BFP^+^ cells. Data are the mean ± SD; *n* = 5 mice per group; **(E)** quantification of the labeling specificity by the percentage of BFP^+^ Cx43^+^ in Cx43^+^ cells. Data are the mean ± SD; *n* = 5 images per group. Yellow scale bar = 1 mm, white scale bar = 100 μm.

We examined the *Cx43-BFP-GFP* reporter expression by fluorescence imaging of a whole mount of organs and immunostaining of frozen organ sections from *Cx43-BFP-GFP* mice at 6 weeks. Although tag BFP reporter expression was not obvious in the whole-mount views (Figures 3A,B), we found that tag BFP was widely expressed in all organs. To further characterize the *Cx43-BFP-GFP* mouse line, BFP and Cx43 were widely expressed in tissues including the heart, liver, lung, stomach, pancreas, brain, kidney, brain, and spleen (Figure 3C). Microscopic quantification showed that 94 ± 0.71% and 94.2 ± 0.58% of BFP^+^ Cx43^+^ express BFP in the heart and brain, respectively (Figure 3D). It is shown that 92.8 ± 0.97% and 81 ± 2.12% of BFP^+^ Cx43^+^ express Cx43 in the heart and brain, respectively (Figure 3E). These data demonstrate that the *Cx43-BFP-GFP* mice line has high labeling specificity and efficiency.

### Characterization of *Cx43-BFP-GFP* reporter line at the embryonic stage

**FIGURE 4:**
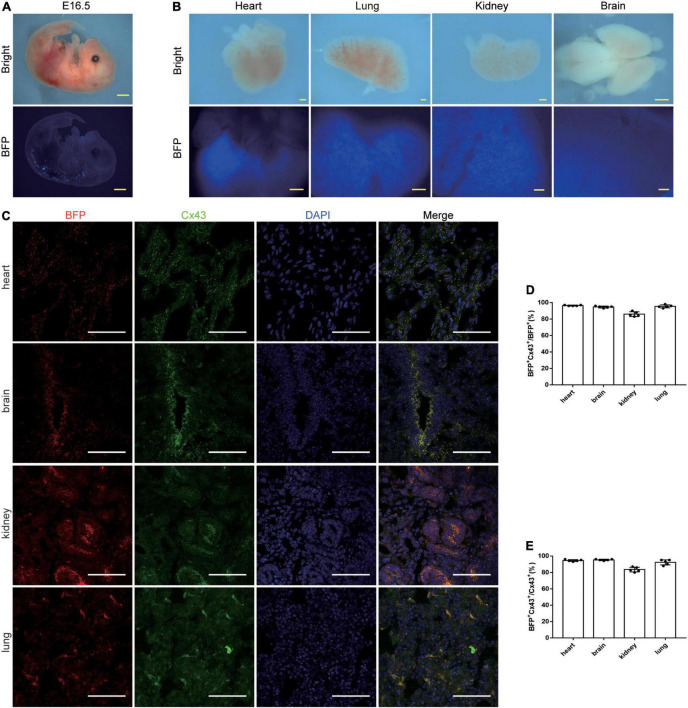
Characterization of *Cx43-BFP-GFP/* + reporter line of E16.5 mice. **(A)** Whole-mount bright field and epifluorescence images of E16.5 *Cx43-BFP-GFP/* + mice; **(B)** whole-mount bright field and epifluorescence images of visceral organs from E16.5 *Cx43-BFP-GFP/* + mice; **(C)** immunostaining for BFP and Cx43 on the sections of several organs. The high magnification images of organs in areas labeled with the square dotted frame, respectively; **(D)** quantification of the labeling efficiency by the percentage of BFP^+^ Cx43^+^ in BFP^+^ cells. Data are the mean ± SD; *n* = 5 images per group; **(E)** quantification of the labeling specificity by the percentage of BFP^+^ Cx43^+^ in Cx43^+^ cells. Data are the mean ± SD; *n* = 5 images per group. Yellow scale bar = 1 mm, white scale bar = 100 μm.

To demonstrate the expression of our tool in embryos, we proceeded to analyze the *Cx43-BFP-GFP* mice at the embryonic stage. At embryonic day (E) 16.5, we detected that BFP signals were robustly expressed in the dorsal neural tube, heart, kidney, lung, and brain but not obvious in other organs from the whole-mount views (Figures 4A,B). Similarly, we immunostained the genetic markers BFP and Cx43 on the tissue sections showing that plenty of BFP^+^ and Cx43^+^ cells were identified in the heart, brain, kidney, and lung at E16.5 (Figure 4C). Microscopic quantification showed that 96.4 ± 0.24% and 94.6 ± 0.51% of BFP^+^ Cx43^+^ express BFP in the heart and brain, respectively (Figure 4D). It is shown that 94.4 ± 0.51% and 95.2 ± 0.37% of BFP^+^ Cx43^+^ express Cx43 in the heart and brain, respectively (Figure 4E). These data indicate that the *Cx43-BFP-GFP* mice line exhibits high labeling efficiency in tissues at the early embryonic stage.

### Expression of Connexin 43 in different cells with *Cx43-BFP-GFP* mice at 6 weeks

**FIGURE 5:**
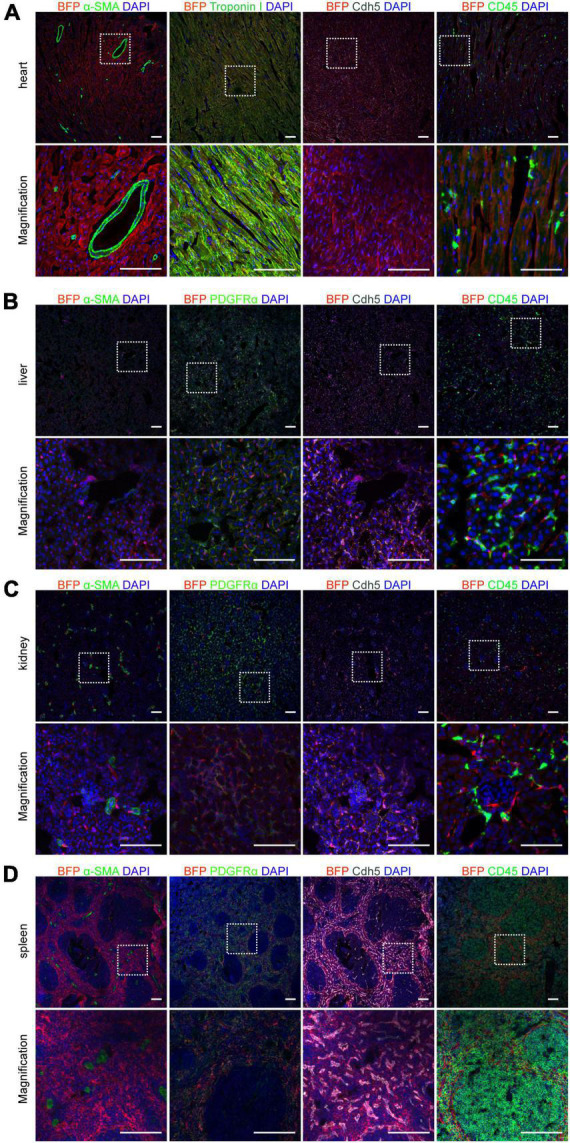
Expression of Cx43 in different cells on 6-week *Cx43-BFP-GFP/* + mice. **(A)** Immunostaining for BFP, Cdh5, CD45, α-SMA, and Troponin I on heart sections; **(B–D)** immunostaining for BFP, α-SMA, PDGFRα, Cdh5, and CD45 on liver, kidney and spleen sections. The high magnification images of organs in areas labeled with the square dotted frame. Scale bar = 100 μm.

Cx43 is widely distributed in various cells of organs throughout the body, especially cardiomyocytes (22). To precisely demonstrate the cell type which expresses BFP reporter in the heart, co-staining assays of heart sections with BFP and either cardiomyocyte marker Troponin I or endothelial cell marker Cdh5 antibodies at 6-weeks *Cx43-BFP-GFP* mice were produced showing that BFP labeled most cardiomyocytes and endothelial cells. However, we did not find BFP expression in coronary vascular SMCs and immune cells, as illustrated in Figure 5A by immunostaining for the smooth muscle cell marker α-SMA and the immune cell marker CD45 on heart sections, consistent with previous reports (23).

In addition, to further assess the expression of Cx43 in cells of various tissues of *Cx43-BFP-GFP* mice at P6W, we performed co-immunostaining for BFP and other cellular markers, including α-SMA, Cdh5, CD45, and the fibroblast marker PDGFRα on other tissue sections showing in Figures 5B–D. The results showed that Cx43 was widely expressed in endothelial cells and fibroblasts in the liver but less expressed in immune cells and smooth muscle cells (Figure 5B). BFP targeted most endothelial cells, immune cells, and fibroblasts on kidney sections. Moreover, our observations confirmed that fewer SMCs were marked by BFP in the adult kidneys, as illustrated in Figure 5C. Similarly, we stained the spleen sections with antibodies against BFP, Cdh5, PDGFRα, or CD45 demonstrating that BFP was detected in many endothelial cells, fibroblasts, and immune cells of the spleen cord. Interestingly, no BFP-labeled SMCs^+^were found in splenic sinuses shown in Figure 5D. We also stained the *Cx43-BFP-GFP* mouse brain, lung, stomach, and thymus with BFP, Cdh5, PDGFRα, and CD45 antibodies to observe the co-localization of BFP and Cx43 in these tissue cells (Supplementary Figure 1), suggesting that BFP reporter expression was confined to specific cells in different organs.

### Connexin 43 protein expression in the hearts of heart failure mice

**FIGURE 6:**
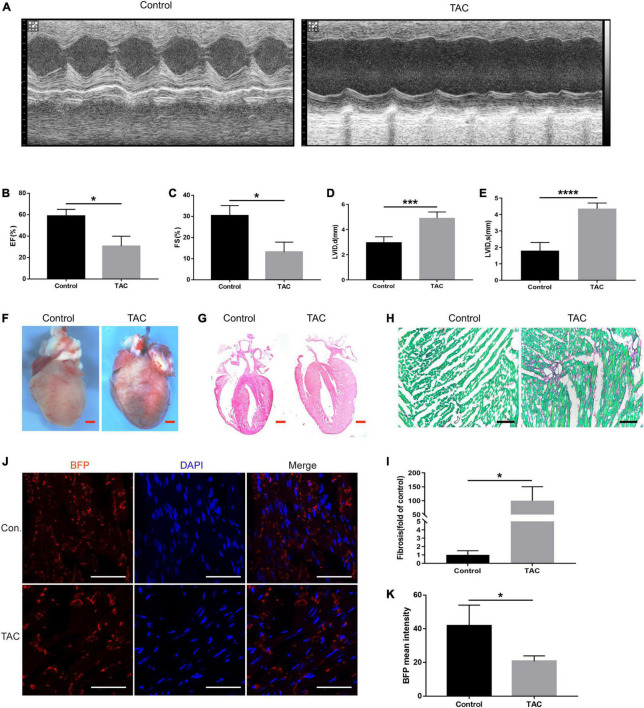
Expression of Cx43 in cardiomyocytes of heart failure. **(A)** Representative M-mode echocardiograms in the control and TAC groups; EF **(B)** and FS **(C)**, LVIDd **(D)**, and LVIDs **(E)** changes in TAC mice evaluated by echocardiography, *P < 0.05, ***P < 0.001, ****P < 0.0001; **(F)** whole-mount bright field images of the heart in two groups; **(G)** HE staining of heart sections of normal mice and TAC-operated mice. **(H,I)** Sirius red staining of the heart sections of normal mice and TAC-operated mice and its quantification analysis, fast green as background color, *P < 0.05; **(J)** expression of BFP (red) in the myocardium of *Cx43-BFP-GFP* mice with heart failure. Red scale bar = 1 mm, black scale bar = 200 μm, white scale bar = 100 μm; **(K)** fading of BFP fluorescent intensity was observed in TAC mice, *P < 0.05.

The murine transverse aortic constriction (TAC) is a commonly used experimental model to mimic AVS and HF (24). So, we evaluated the cardiac function of the mice by echocardiography 4 weeks after TAC in *Cx43-BFP-GFP* mice. Figure 6A displays representative M-mode echocardiograms showing evidence of HF in TAC group. Similarly, we evaluated the EF, FS, and LVID in mice, as shown in Figures 6B–E. The results showed that EF and FS were significantly reduced, LVIDd, and LVIDs increased significantly in TAC mice. TAC mice had markedly enlarged hearts and massive collagen fibril deposition in the myocardium (Figures 6F–H). The fibrosis area in the HF mice was approximately 100 times higher than that of the control ones (Figure 6I). Similarly, we stained myocardial tissues with the fluorescent marker BFP and found that the expression of Cx43 was widely and uniformly distributed in normal myocardial tissue; however, the fluorescence expression of Cx43 is decreased and distributed unevenly in the myocardial tissues of HF mice (Figures 6J,K). The co-staining of BFP and other cell markers could further clarify the main cellular transformation of Cx43 in the decompensation of intercellular signaling in cardiomyopathy caused by valvular disease, which is beneficial to the subsequent determination of the mechanism of HF caused by other factors such as valvular disease.

## Conclusion

In our study, we found that AVS disease, which is an independent factor for HF, had marked fibrosis and uneven distribution of GJs in the myocardium. To better explore this pathological process, we established the *Cx43-BFP-GFP* mouse model. In this reporter line, Cx43^+^ cells can be labeled with BFP, with high labeling efficiency in important tissues and organs, especially cardiac cells. The efficacy of this reporter line has been validated in the TAC HF model with decreased expression and uneven distribution of Cx43 in cardiomyocytes of mice with HF. This dual reporter gene line provides a unique genetic tool for understanding the pathological process of myocardial remodeling and provides a new treatment direction for delaying HF.

## Discussion

Various factors that cause myocardial injury may lead to cardiac systolic dysfunctions, resulting in HF. Among them, the pathological injury of AVS is one of the leading factors (25–27). Previous reports have found that moderate aortic stenosis was related to poor prognosis in patients with HFrEF (28). The myocardium of valvular stenosis has obvious fibrosis. This process involves the participation of a variety of cells, among which the coupling and signal transduction mechanisms between cardiomyocytes, endothelial cells, fibroblasts, and other cells are not fully understood yet. Previous studies pointed out that the Wnt signaling pathway had an important role in the pathogenesis of HF. The Wnt signaling pathway is mainly transduced in cells through two different pathways, the canonical Wnt/β-catenin pathway and the non-canonical Wnt/Ca^2+^ pathway (29). Persistent activation of endothelial Wnt/β-catenin signaling induces HF (30). Besides, cardiac fibroblasts are the main cell types in cardiac fibrosis (31); interestingly, Wnt/β-catenin signaling also had vital functions in regulating fibroblast activation and ECM gene expressions (32). Thus, studying the Wnt/β-catenin pathway is the key to unveiling the pathogenesis in HF.

Cx43 is considered to be a target of Wnt/β-catenin transcription, which interacts with the β-catenin (33, 34). One of the critical mechanisms of cardiac remodeling is the change in intracellular electrical coupling caused by aberrant expression of Cx43 protein (35, 36). Moreover, its ubiquitous expression at the poles of ventricular and atrial myocytes and in certain regions of the conduction system (22, 37) has significantly correlated with cardiac arrhythmia. Thus, the observation of Cx43 *in vivo* is beneficial to a better understanding of the pathogenetic mechanism and is essential for drug screening and other clinical practices in HF.

Several Cx43 Cre-loxp and Cx43 Cre-ER (T)/+ (38, 39) mouse tools were generated previously by transgene and applied to study diseases such as arrhythmia (40). Cx43kiECFP (41) and Cx43kiLacZ (42) mouse tools have been used in specific subsets of cells. The advantage of the *Cx43-BFP-GFP* line developed in this study is that Cx43^+^ cells can be labeled with BFP in a natural state, and the GFP could be expressed by crossing the specific Cre mice if available. Therefore, the dual reporter system is expected to explore further the interaction and transformation of cardiac fibroblasts, endothelial cells, cardiomyocytes, and other cells in HF or other heart-related diseases.

Our research has introduced a double-reporter *Cx43-BFP-GFP* line. This mouse line cannot only display the existence of Cx43 but also be used as a conditional knockout line with the second reporter of eGFP. Meanwhile, to date, the experiments from *Cx43-BFP-GFP*: Cre mice remain lacking. Crossing the reporter line with Cre mice to verify the advantage of the dual reporter system in HF still needs further research.

## Data availability statement

The original contributions presented in this study are included in the article/Supplementary material, further inquiries can be directed to the corresponding author/s.

## Ethics statement

The studies involving human participants were reviewed and approved by the Ethics Committee of Zhongshan Hospital, Affiliated With Fudan University. The patients/participants provided their written informed consent to participate in this study. The animal study was reviewed and approved by the Ethics Committees of Zhongshan Hospital, Affiliated With Fudan University.

## Author contributions

SL and YL performed material preparation, data collection, and analyses. QZ and YZ edited and commented on the manuscript. KL and TL bred the mice, performed the experiments, and analyzed the data. JG supervised the study and analyzed the data. YY contributed to the study conception and design. All authors reviewed the manuscript and agreed to be accountable for the content of the work.
