# Supplementary material for: Expression of connexin 43 protein in cardiomyocytes of heart failure mouse model
Source: Front Cardiovasc Med. 2022 Oct 5;9:1028558. doi: 10.3389/fcvm.2022.1028558 (PMC9581147; doi:10.3389/fcvm.2022.1028558)
Supplement: Supplementary file 1 [file Data_Sheet_1.docx]

**Supplementary Figure 1 Expression of Cx43 in different cells on 6-weeks *Cx43-BFP-GFP/+* mice.**

**(A)** Immunostaining for BFP, PDGFRα and CD45 on brain sections; **(B-D)** Immunostaining for BFP, α-SMA, PDGFRα, Cdh5 and CD45 on lung, stomach and thymus sections. The high magnification images of organs in areas labeled with square dotted frame. Scale bar = 100 μm.
