# Supplementary material for: Expression of connexin 43 protein in cardiomyocytes of heart failure mouse model
Source: Front Cardiovasc Med. 2022 Oct 5;9:1028558. doi: 10.3389/fcvm.2022.1028558 (PMC9581147; doi:10.3389/fcvm.2022.1028558)
Supplement: Supplementary file 2 [file Table_1.docx]

**Supplement table1 Information of patients and donors**

| **NO.** | 1 | 2 | 3 |
| --- | --- | --- | --- |
| **Gender** | F | F | F |
| **Age** | 64 | 74 | 72 |
| **Diagnosis** | AVS | AVS | AD |
| **Degree of stenosis** | severe | severe | normal |
| **NYHA** | III | IV | II |
| **LVEF(%)** | 63 | 58 | 61 |
| **Hypertension** | - | - | - |
| **Diabetes** | - | - | - |
| **Rheumatism** | - | - | - |
