# Supplementary material for: Expression of connexin 43 protein in cardiomyocytes of heart failure mouse model
Source: Front Cardiovasc Med. 2022 Oct 5;9:1028558. doi: 10.3389/fcvm.2022.1028558 (PMC9581147; doi:10.3389/fcvm.2022.1028558)
Supplement: Supplementary file 3 [file Table_2.docx]

**Supplement table 2 List of Abbreviations**

| ****Abbreviation**** | **Full name** |
| --- | --- |
| HF | Heart failure |
| Cx43 | Connexin 43 |
| AVS | Aortic valve stenosis |
| HFrEF | Heart failure with reduced ejection fraction |
| AVR | Aortic valve replacement |
| GJ | Gap junction |
| Gja1 | Gap junction protein, Alpha 1 |
| HE | Hematoxylin-esosin |
| EF | Ejection fraction |
| FS | Fraction shortening |
| LVID,d | Left ventricular internal diameter at end-diastole |
| LVID,s | Left ventricular internal diameter at end-systole |
| TAC | Transverse aortic constriction |
