# Supplementary figures and images for: Expression of connexin 43 protein in cardiomyocytes of heart failure mouse model
Source: Front Cardiovasc Med. 2022 Oct 5;9:1028558. doi: 10.3389/fcvm.2022.1028558 (PMC9581147; doi:10.3389/fcvm.2022.1028558)

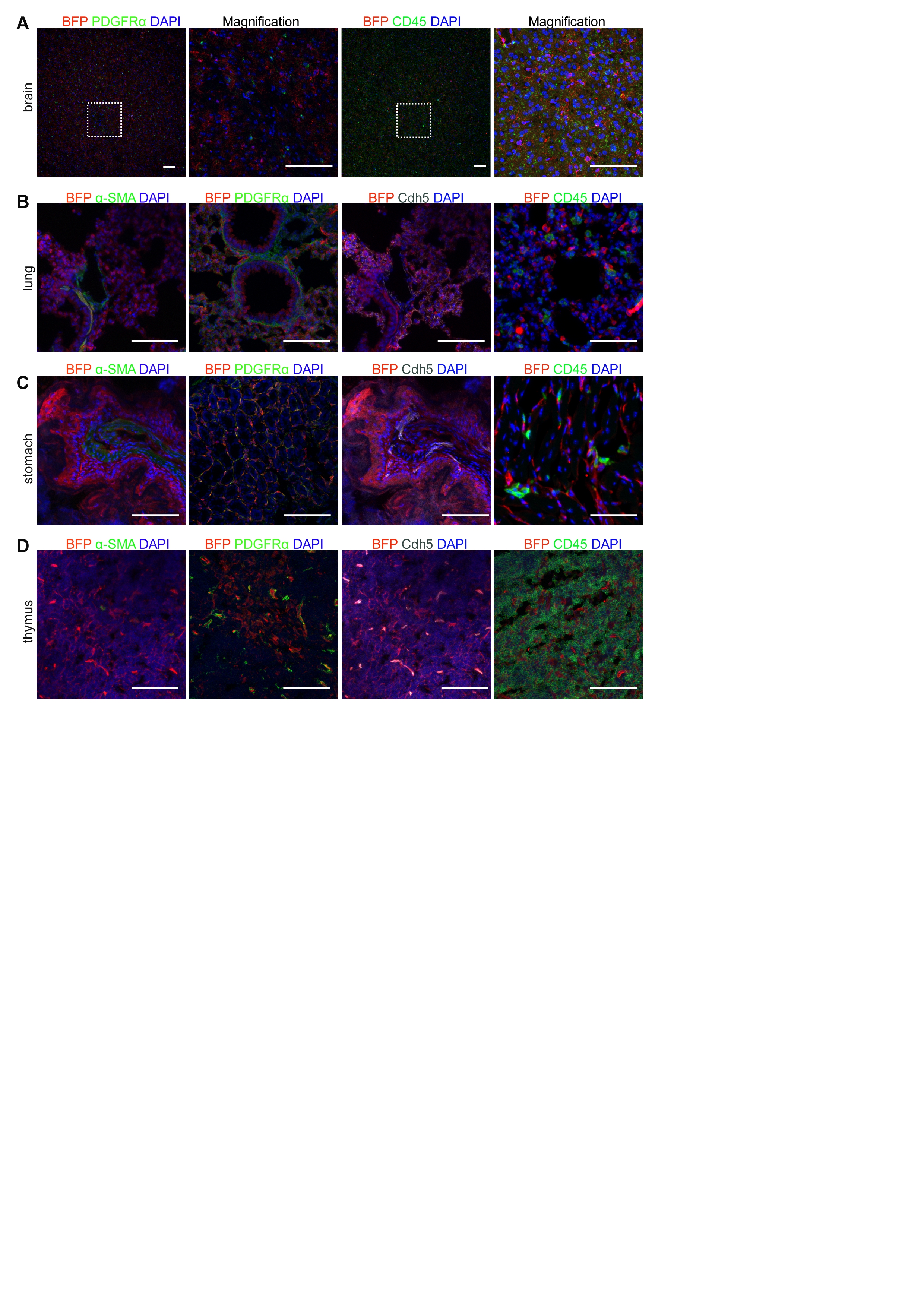

Supplement: Supplementary file 4 [file Image_1.JPEG]
